# Automated data preparation for *in vivo* tumor characterization with machine learning

**DOI:** 10.3389/fonc.2022.1017911

**Published:** 2022-10-11

**Authors:** Denis Krajnc, Clemens P. Spielvogel, Marko Grahovac, Boglarka Ecsedi, Sazan Rasul, Nina Poetsch, Tatjana Traub-Weidinger, Alexander R. Haug, Zsombor Ritter, Hussain Alizadeh, Marcus Hacker, Thomas Beyer, Laszlo Papp

**Affiliations:** ^1^ QIMP Team, Center for Medical Physics and Biomedical Engineering, Medical University of Vienna, Vienna, Austria; ^2^ Department of Biomedical Imaging and Image-guided Therapy, Division of Nuclear Medicine, Medical University of Vienna, Vienna, Austria; ^3^ Christian Doppler Laboratory for Applied Metabolomics, Medical University of Vienna, Vienna, Austria; ^4^ Department of Medical Imaging, University of Pécs, Medical School, Pécs, Hungary; ^5^ 1st Department of Internal Medicine, University of Pécs, Medical School, Pécs, Hungary; ^6^ Applied Quantum Computing group, Center for Medical Physics and Biomedical Engineering, Medical University of Vienna, Vienna, Austria

**Keywords:** cancer, hybrid imaging, PET, data preprocessing, machine learning

## Abstract

**Background:**

This study proposes machine learning-driven data preparation (MLDP) for optimal data preparation (DP) prior to building prediction models for cancer cohorts.

**Methods:**

A collection of well-established DP methods were incorporated for building the DP pipelines for various clinical cohorts prior to machine learning. Evolutionary algorithm principles combined with hyperparameter optimization were employed to iteratively select the best fitting subset of data preparation algorithms for the given dataset. The proposed method was validated for glioma and prostate single center cohorts by 100-fold Monte Carlo (MC) cross-validation scheme with 80-20% training-validation split ratio. In addition, a dual-center diffuse large B-cell lymphoma (DLBCL) cohort was utilized with Center 1 as training and Center 2 as independent validation datasets to predict cohort-specific clinical endpoints. Five machine learning (ML) classifiers were employed for building prediction models across all analyzed cohorts. Predictive performance was estimated by confusion matrix analytics over the validation sets of each cohort. The performance of each model with and without MLDP, as well as with manually-defined DP were compared in each of the four cohorts.

**Results:**

Sixteen of twenty established predictive models demonstrated area under the receiver operator characteristics curve (AUC) performance increase utilizing the MLDP. The MLDP resulted in the highest performance increase for random forest (RF) (+0.16 AUC) and support vector machine (SVM) (+0.13 AUC) model schemes for predicting 36-months survival in the glioma cohort. Single center cohorts resulted in complex (6-7 DP steps) DP pipelines, with a high occurrence of outlier detection, feature selection and synthetic majority oversampling technique (SMOTE). In contrast, the optimal DP pipeline for the dual-center DLBCL cohort only included outlier detection and SMOTE DP steps.

**Conclusions:**

This study demonstrates that data preparation prior to ML prediction model building in cancer cohorts shall be ML-driven itself, yielding optimal prediction models in both single and multi-centric settings.

## Introduction

Cancer is the leading cause of death worldwide, accounting for approximately 10 million death cases in 2020 (1). Molecular and hybrid imaging have a prominent role in cancer detection, diagnosis and evaluation by assessing physiological aspects on a molecular level non-invasively (2, 3). Hybrid imaging provides both morphological and functional information of patients, as well as the assessment of quantitative information for tumor characterization (4), however, it is mainly used for visual assessment in the clinical routine. In contrast, recent studies have demonstrated the added value of radiomics to analyze tumors directly in imaging data. As such, radiomics was shown to predict clinical endpoints, such as survival, risk assessment, treatment response as well as to characterize tumor heterogeneity (5–7). Here, the Imaging Biomarker Standardization Initiative (IBSI) has been aiding to execute and report radiomics analyses in a standardized way in order to support repeatability of derived features (8). Once established, radiomics readouts can be used in combinations with machine learning (ML) approaches to establish high performing predictive models (9–14). Due to the low sample count as a natural characteristic of hybrid imaging datasets, classical ML approaches are preferred over deep learning (DL) algorithms that demand large scale input data for model training (15–17).

Nevertheless, radiomic studies routinely encounter challenges, such as high feature counts (sparse feature spaces for ML) as well as high feature redundancy when combined with ML approaches (18, 19). In addition, the presence of outliers or borderline cases may further affect the performance of ML prediction models (20, 21). Last, class imbalance, originating from sparse occurrence of various disease subtypes further influences ML predictive performance, where minority subtypes can be systematically misclassified (22, 23). The above properties are representative in cancer cohorts. Therefore, data preparation is increasingly becoming a necessity in radiomic studies combined with ML approaches to build oncological prediction models (24–26).

Data preparation refers to various methods that are performed prior to ML to optimize the training data for e.g., subclass imbalance correction, outlier handling, as well as feature selection and dimensionality reduction. While data preparation remains underrepresented in the field of hybrid imaging ML analysis, it has been estimated that approximately 70% of workload is spent with manual data preparation prior to ML in industry environments driven by ML (27, 28).

Since determining the ideal configuration of data preparation is a complex and time-consuming process, we hypothesize that it shall become ML-driven on its own, thus, maximizing model performance in various cancer cohorts and significantly reducing the time for the creation of ML workflows. Therefore, the objectives of this study were: (a) to propose an ML-driven data preparation (MLDP) approach which automatically selects consecutive data preparation algorithms and their hyperparameters for defining a data preparation pipeline prior to ML-based prediction modelling. (b) to estimate the added value of MLDP in various ML predictive models, comparing their respective predictive performance with and without MLDP, as well as with manual preparation.

## Materials and methods

### Data collection

**Figure 1:**
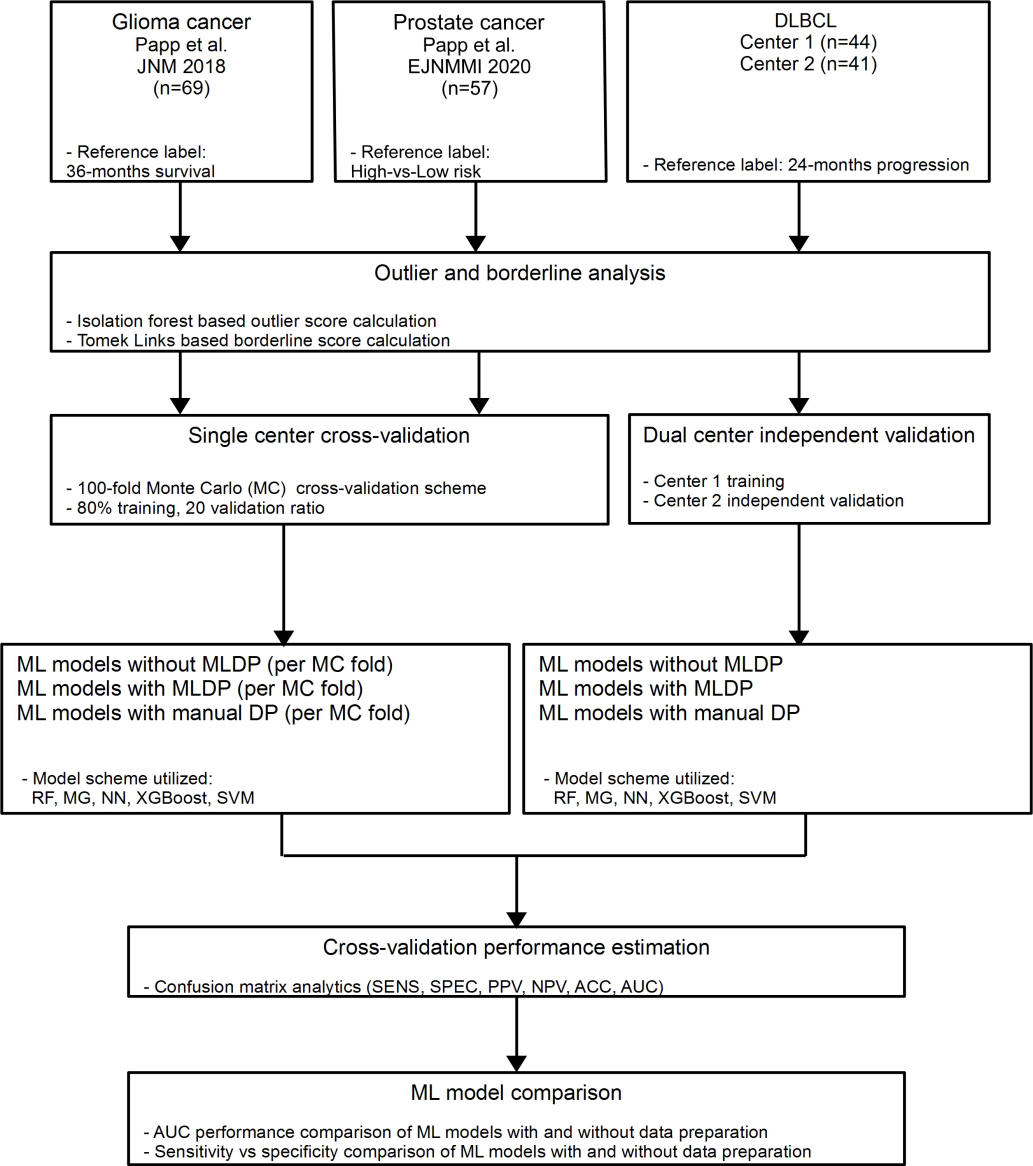
In this study, two single center glioma (29) and prostate (10) cancer and one dual-center diffuse large B-cell lymphoma (DLBCL) (30) cohorts were analyzed retrospectively. Outlier and borderline scores were calculated for all cohorts. For single center data, 100-fold Monte Carlo (MC) cross-validation scheme was utilized with 80%-20% training/validation data split. For dual center DLBCL analysis, Center 1 dataset was used for training and Center 2 for independent validation. Predictive models were established with and without machine learning-driven data preparation (MLDP) per training-validation pair in each cohort. All model building utilized five machine learning (ML) schemes: Random Forest (RF), Multi Gaussian (MG), Extreme gradient boosting (XGBoost), Neural networks (NN) and support vector machine (SVM). Predictive performance of each model scheme was evaluated with confusion matrix analytics. Performance comparison of ML models with and without incorporated MLDP was conducted for each analyzed cohort. DLBCL, Diffuse large B-cell lymphoma; ACC, Accuracy; SNS, Sensitivity; SPC, Specificity; PPV, Positive predictive value; NPV, Negative predictive value; AUC, Area under the receiver operator characteristics curve.

In this study, three clinically relevant cancer cohorts were included retrospectively to investigate the added value of ML-driven data preparation (MLDP) (**Figure 1**). Written informed consent was obtained from all patients before examination and their respective studies were approved by their local institutional review boards (10, 29, 30). The cohort selection process focused on collecting cancer imaging cohorts of various imaging systems, tracers, sample sizes, feature counts and subclass imbalance ratios. In addition, all datasets were composed of radiomic features coming from different imaging modalities (**Table 1**). Two out of three analyzed cohorts originated from a single center-only, and one originated from two centers. See **Figure 1** for the CONSORT diagram of the study. For the Imaging Biomarker Standardization Initiative (IBSI)-conform extracted radiomic features of each involved radiomic study, see their respective references.

**Table 1:** Characteristics of cancer cohorts used in study.

Cohort	Prediction	Centers	Data	Samples	Features	Imbalance ratio [%]	Reference
Glioma	36-months survival	Single	^11^C-MET PET	69	160	67-vs-33	(29)
Prostate cancer	high-vs-low risk	Single	^68^GA-PSMA PET/MRI	57	306	52-vs-48	(10)
DLBCL Center 1	24-months progression	Dual	^18^F-FDG PET/CT	44	57	32-vs-68	(30)
DLBCL Center 2				41		39-vs-61	

MET, Methionine; PET, Positron Emission Tomography; FNA, Fine Needle Aspiration; PSMA, Prostate specific membrane antigen; MRI, Magnetic resonance imaging; CT, Computed Tomography; DLBCL, Diffuse large B-cell lymphoma.

### Dataset characteristics analysis

Outlier and borderline scores were calculated to estimate the presence of outliers and borderline samples within the analyzed datasets. The isolation forest (31) method was utilized for outlier detection. To determine the outlier score, the percentage of detected outliers was calculated with respect to total sample count. For borderline score calculation, Tomek Links (32) was utilized including the minority subclass samples. The percentage of the minority borderline samples presence was then calculated with respect to total sample count. See Supplemental S1 for hyper-parameters of the utilized algorithms for both outlier and borderline score calculations.

### Data preparation methods

In this study, various, well established, data pre-processing methods were incorporated to perform data preparation prior to machine learning. Synthetic minority oversampling technique (SMOTE) (33), borderline synthetic minority oversampling technique (BSMOTE) (34), and random oversampling method (35) were employed to handle subclass imbalance correction. Tomek link (32) approach was utilized for data purification. Isolation forest (31) was employed for outlier detection and elimination. R-squared based sequential forward selection (SFS) (36) was employed to perform feature selection and principal component analysis (PCA) (37) was incorporated to reduce high number of dimensions with data transformation approach.

### Data preparation pipelines

Data preparation pipelines – containing an ordered list of data preparation steps – were defined for each cohort automatically (see Sec. ML-driven data preparation). To guide and regulate this process, this study defined restrictions to build data preparation pipelines. Restrictions covered the range of maximum number of methods allowed in a pipeline, no repetitions of the same method and restrictions regarding co-existence of certain method pairs in each pipeline. For details of these restrictions and the way of building pipelines see Supplemental S2.

### ML-driven data preparation

**Figure 2:**
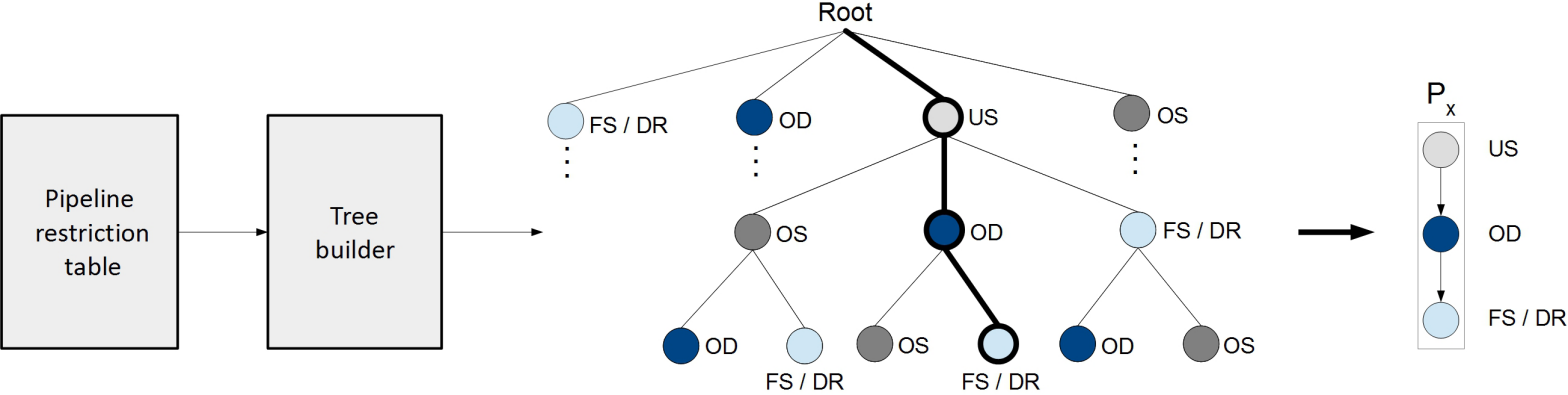
Pipeline tree generation based on the pre-determined restriction conditions. The pipeline restriction table contains rules regarding permitted, consecutive data preparation steps and the permitted number of repetitions of particular data preparation methods. The Tree builder generates a tree of all possible data preparation pipelines, satisfying the rules defined in the Pipeline restriction table. A particular pipeline is defined as the ordered steps of data preparation methods from tree root to any leaf. P_x_, randomly selected data preparation pipeline; FS, Feature selection; DR, Dimensionality reduction; OD, Outlier detection; US, Undersampling; OS, Oversampling.

This study utilized machine learning approaches to identify the optimal data preparation pipeline for each of its input cohorts, where receiver operator characteristics (ROC) distance fitness is measured strictly over the training dataset (29). The validation dataset was not involved in any decision making processes. For this purpose, all possible pipeline variants that satisfy the defined restrictions were pre-generated and stored in a pipeline tree per cohort (**Figure 2**). The tree contains pipelines with identical preparation algorithms included but in different sequential order, thus allowing the MLDP to evaluate the importance of preparation steps ordering within a pipeline. The machine learning approach to build data preparation pipelines utilized evolutionary principles (38–40) to iteratively select pipeline variant pairs from the pipeline tree, followed by generating a new offspring pipeline from them, which also satisfies the pipeline restrictions. In addition, hyperparameter optimization of established pipelines was also performed (see Supplemental S3 and S6). This approach naturally converged towards an optimal pipeline. See Supplemental S3 for details of the evolutionary algorithm.

### Predictive performance estimation

**Figure 3:**
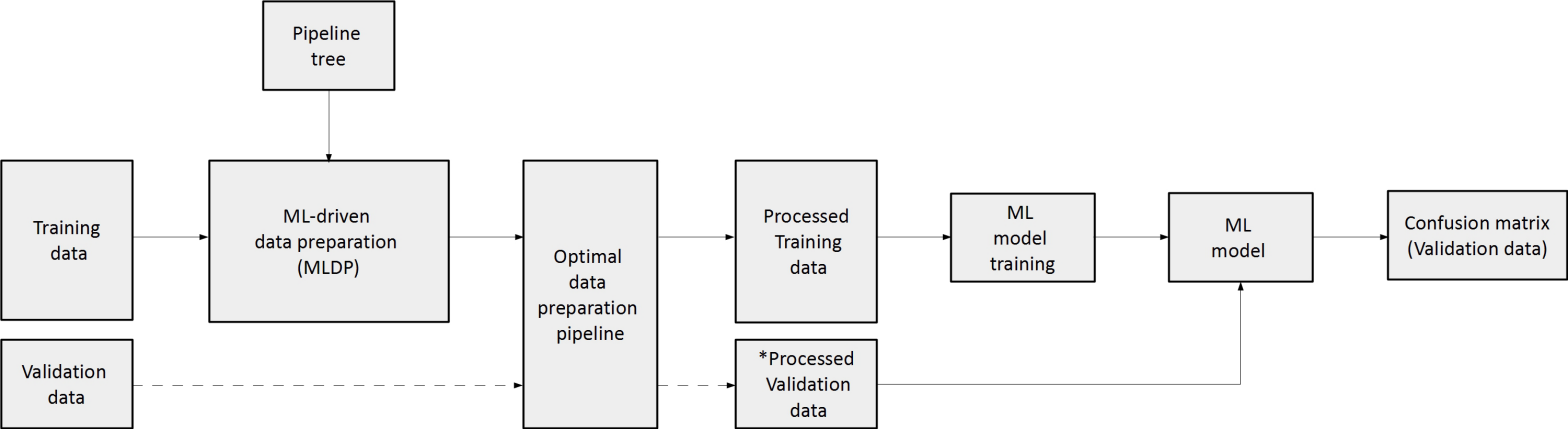
The concept of machine learning-driven data preparation (MLDP). Given a training and validation subset pair, the training subset is the input of MLDP, which has access to the pipeline tree (**Figure 2**). The MLDP identifies an optimal data preparation pipeline from the tree by solely analyzing the training subset. The preprocessed training dataset is the input for machine learning (ML) to build the prediction model. Data preparation algorithms that operate in the feature space are also applied to the validation subset (e.g., feature selection). The preprocessed validation subsets serve as inputs to the built ML prediction model to estimate cross-validation performance of the given model. ML – Machine learning; * - only preparation steps, which operate in feature space (feature selection/dimensionality reduction) are applied on validation data.

To estimate the performance of the evolutionary algorithm for pipeline building, 100-fold Monte Carlo (MC) cross-validation with training-validation split of 80-20% was utilized for single-center cohorts, which assures lower data variance due to the high iteration count compared to other suggested cross-validation methods such as Leave One Out (LOO) (41). In case of the dual-centric cohort, Center 1 and Center 2 was chosen to act as a training set and independent validation set respectively. The evolutionary algorithm utilized solely the given training dataset to build an optimal data preparation pipeline, thus, risk of overfitting the model was minimized. The ML predictive model was established on the preprocessed training dataset (**Figure 3**). In order to estimate machine learning method bias, this study built five different machine learning models for each preprocessed training set utilizing random forest (RF) (42), multi-gaussian (MG) (29), support vector machine (SVM) (43), extreme gradient boosting (XGBoost) (44) and neural networks (NN) (45).

To estimate the performance of the MLDP-trained pipeline in each Monte Carlo fold, the respective validation dataset was processed by its methods that operate in the feature space (e.g., feature selection and dimensionality reduction). The reason of not executing the whole pipeline on the validation set was that some steps, such as SMOTE are operating in sample space and hence, shall only be applied on the training set (25). The processed validation cases were inputs of the RF, SVM, XGBoost, NN and MG model variants per cross-validation fold. Predictive performance estimation across 100-folds for single-center studies and across Center 2 independent validation cases of the dual-center study were done by confusion matrix analytics (24), where accuracy (ACC), sensitivity (SENS), specificity (SPEC), positive predictive value (PPV), negative predictive value (NPV) and area under the receiver operator characteristics curve (AUC) were calculated per model variant across validation samples. The significance of ML predictive model performance with and without MLDP was analyzed with ANOVA test (Microsoft Excel 2016), resulting in dedicated p-values, where p<0.05 was considered as significance threshold. The analysis was conducted over validation results across all MC folds for each predictive model. In addition, mean, standard deviation and confidence interval (CI 95%) were calculated. In order to compare the performance of MLDP to manual preparation, a fixed data preparation pipeline including feature selection and SMOTE algorithms was incorporated. The choice of data preparation steps was based on the literature review of similar works that addressed the high feature dimensionality of radiomic datasets and the highly imbalanced nature of diseases they attempted to characterize (13, 46, 47). In addition, comparisons without MLDP or any preprocessing were also performed. In latter case, the datasets were not modified at all but taken for analysis on an as-is basis.

## Results

### Dataset characteristics analysis

The outlier score calculations revealed low outlier presence across all analyzed cohorts (0.0% - 2.4%). The Center 1 cohort from the DLBCL dataset presented outliers (2.4%), while Center 2 had no outliers present (0.0%). The average borderline score across all cohorts were 25.7% (21.4% - 29.6%). See **Table 2** for outlier and borderline score calculations across all cohorts.

**Table 2:** Outlier and borderline score ratio across the MC folds of each analyzed cohort.

	Prostate cancer	Glioma	DLBCL Center 1	DLBCL Center 2
Outlier score	0.5	1.3	2.4	0.0
Borderline score	29.6	21.4	29.3	25.0

DLBCL, Diffuse large B-cell lymphoma. Scores are expressed in percentages [%].

### ML-driven data preparation

Analysis of data preparation method occurrences per 100 MC cross-validation folds revealed high presence of the outlier detection algorithm (IF) in pipelines with 70% - 80% occurrence in single-center cohorts. In the dual-center cohort, both IF and SMOTE were present in the optimal pipeline as provided by the MLDP. Furthermore, SMOTE yielded high presence in single-center data preparation pipelines (39% - 50%) as well as the R-Squared feature selection method (38% - 82%). Random undersampling occurred moderately (30% - 35%). Borderline SMOTE and Tomek Links showed minor impact with 0% - 4% and 0% - 14% occurrences, respectively. See **Table 3** for detailed description of data preparation pipelines across the 100 Monte Carlo cross-validation folds for all cohorts.

**Table 3:** Data preparation method occurrences across the 100 Monte Carlo cross-validation folds per cohort.

**Cohort**	**OD**	**FS**	**RO**	**RU**	**SMOTE**	**BSMOTE**	**TL**
Prostate	70	38	9	35	39	4	8
Glioma	80	82	5	30	50	0	14
DLBCL	100*	0	0	0	100*	0	0

Note that non-zero occurrences of DP steps do not imply that they were mutually present in particular pipelines. Method occurrence is shown in percentage [%]. OD, Outlier detection; FS, Feature selection; RO, Random oversampling; RU, Random undersampling; SMOTE, Synthetic minority oversampling technique; BSMOTE, Borderline synthetic minority oversampling technique; TL, Tomek Links.

Cell color codes demonstrate data preparation occurrences within the range of 0% to 100% with bright and dark colors respectively. * - occurrence in multi-center data analysis is always 100% due to independent validation (single run – no cross-validation).

Established data preparation pipelines showed high complexities in preparation the prostate cancer, and glioma cohorts, by incorporating high numbers of data preparation methods (n=6-7). In contrast, the pipelines for preparing the training data for DLBCL machine learning analysis consisted of only two methods (**Table 3**).

### Predictive performance estimation

The random forest (RF) model scheme achieved the highest performance increase of +0.16 AUC (p<0.001) with MLDP (0.87 AUC) compared to no MLDP (0.71 AUC) for predicting 36-months survival in the glioma cohort. Similarly, the SVM demonstrated a +0.13 AUC (p<0.001) increase with MLDP (0.86 AUC) compared to no MLDP (0.73 AUC) in the same cohort. In contrast, MG demonstrated the lowest performance increase of +0.01 AUC with MLDP (0.75 AUC). On average, the RF and NN models benefited the most with the average performance increase of +0.06 AUC across all cohorts, while XGBoost model demonstrated the least performance increase of +0.01 AUC. Average performance increase across all ML approaches was +0.05 AUC while utilizing MLDP.

In addition, ML schemes with utilizing MLDP outperformed the manual data preparation-based models across all cohorts, except for predicting 36-months survival in the glioma cohort where the NN model benefited equally from both data preparation approaches (0.80 AUC).

On average, the highest increase of +0.09 AUC was achieved in the glioma cohort across all ML methods when utilizing MLDP (0.85 AUC). In contrast, ML models for prostate cancer high-vs-low risk prediction benefited the least from utilizing MLDP with +0.01 AUC (0.78 AUC). See **Table 4** for the cross-validation AUC of all cohorts and ML methods with and without MLDP, with manual data preparation, as well as for their respective p-values.

**Table 4:** The cross-validation area under the receiver operator characteristics curve (AUC) of all cohorts and machine learning (ML) methods with and without ML-driven data preparation (MLDP) as well as manually preprocessed across Monte (MC) cross-validation folds.

Classifier	MLDP	Prostate		Glioma		DLBCL		Average
		AUC	P-value	AUC	P-value	AUC	P-value	
RF	No	0.77	0.002	0.71	<0.001	0.76	NA*	0.75
	Yes	0.79		0.87		0.78		0.81
	Manual	0.77		0.79		0.76		0.77
MG	No	0.74	0.496	0.73	<0.001	0.68	NA*	0.72
	Yes	0.75		0.83		0.74		0.77
	Manual	0.74		0.82		0.73		0.73
XGBoost	No	0.79	0.244	0.88	0.028	0.67	NA*	0.78
	Yes	0.79		0.92		0.67		0.79
	Manual	0.75		0.84		0.67		0.75
NN	No	0.78	0.761	0.71	<0.001	0.55	NA*	0.68
	Yes	0.78		0.80		0.63		0.74
	Manual	0.73		0.80		0.61		0.71
SVM	No	0.77	0.517	0.75	<0.001	0.63	NA*	0.72
	Yes	0.77		0.85		0.69		0.77
	Manual	0.76		0.82		0.67		0.75
Average	No	0.77	0.404	0.76	<0.001	0.66	NA*	0.05
	Yes	0.78		0.85		0.70	
	Manual	0.75		0.81		0.69	

RF, Random Forest; MG, Multi gaussian; XGBoost, Extreme gradient boosting; NN, Neural networks; Cell color codes demonstrates the increased/unchanged performance of ML models combined with. In addition, p-values of compared predictive models (with and without MLDP) are included.

Models with unchanged AUC performance for predicting high-vs-low risk in prostate cancer cohort still demonstrated a more balanced sensitivity (SENS) and specificity (SPEC) with MLDP. Utilizing MLDP, the NN model yielded 0.77% SENS and 0.78% SPEC, respectively, compared to 0.83% SENS and 0.71% SPEC without MLDP. Similarly, the SVM model yielded 0.76% SENS and 0.78% SPEC with MLDP compared to no MLDP (0.80% SENS, 0.74% SPEC). See Supplemental S4 for sensitivity, specificity, positive predictive value, negative predictive value and accuracy cross-validation values across each model and cohort. For detailed information about conventional statistical analysis such mean, standard deviation, confidence interval (CI 95%) and p-values of each established model see Supplemental S5.

## Discussion

In this study we proposed a machine learning-driven data preparation approach (MLDP) to automate the building process of data preparation pipelines prior to building ML prediction models for radiomic studies. We investigated the effects of the proposed approach on machine learning predictive performance across various single and dual-center cancer cohorts and achieved up-to +0.16 AUC increase compared to performing no data preparation and up to +0.08 AUC com-pared to manually performed data preparation.

Across all ML approaches, the prediction models established for glioma cohort benefited the most from MLDP (+0.09 AUC) compared to +0.05 AUC from manual DP, while the models for prostate cohort did not significantly benefit from it. This is in line with their respective imbalance ratios (0.33 for glioma vs. 0.48 for prostate), implying, that class imbalance – even with utilizing imbalance correction approaches such as SMOTE – has the most-influential effect on ML prediction. The above findings are logical, given that most disease subtypes have an imbalanced occurrence (10, 13, 24, 25, 46–48). The RF and NN methods demonstrated the highest average AUC increase of +0.06 across all cohorts, while XGBoost yielded the lowest AUC increase of average +0.01. Nevertheless, XGBoost demonstrated a relatively high performance compared to other ML approaches before applying MLDP. We assume that this is due to the guided training process of XGBoost, also referred to as gradient boosting (44). The highest average AUC of 0.85 across all cohorts was achieved by the RF models when utilizing MLDP.

MLDP pipelines that increased overall performance the most, were also the most complex, containing an average of 6-7 data preparation steps. The highest occurrence of data preparation steps across single-center MC folds were outlier detection (70% - 80%), imbalance correction (SMOTE) (39% - 50%) and feature selection (38% - 82%) methods. On the other hand, MLDP only included two data preparation steps (OD and SMOTE) in the dual-center cohort. We consider that the high data preparation step count in single center studies may be due to the chosen cross-validation scheme. Monte Carlo cross-validation performs a random split to generate a training and a validation subset. It is one of the preferred cross-validation approaches, since it minimizes the risk of training subset selection bias (49). Nevertheless, selected splits may result in training-validation feature value distributions that are less similar compared to distributions of a dual, or multi-center dataset which represents reality. While this phenomenon appears suboptimal, it is one of the best practices to avoid overestimating single-center prediction model performance (4). Since MLDP was utilized per-training split, we assume that the high preparation step count is the result of attempting to counter-balance the above effect.

Single-center machine learning studies even with utilizing cross-validation may tend to over-estimate performance because of the bias in the data itself (49). Therefore, the characteristics of MLDP outputs shall not be interpreted solely by single-center investigations. Nevertheless, our dual-center cohort analysis successfully demonstrated the expected behavior of MLDP. Specifically, the Center 1 dataset had detectable outliers (2.4%) in the DLBCL dual-center cohort. The optimal data preparation steps as built by the MLDP contained outlier detection (OD) as a necessary step for preparation Center 1 prior to machine learning. The dual-center DLBCL dataset originated from the same country and region representing similar cohort characteristics, even though the imaging data came from two different scanner types. Consistently, the DLBCL optimal pipeline contained only two data preparation steps that were sufficient to yield high-performing prediction models in this cohort. Nevertheless, feature distribution similarities in case of multi-national and/or multi-centric datasets is not guaranteed and, thus, may require a more complex data preparation pipeline.

Even though borderline score calculations yielded relatively high presence across all cohorts (21% – 30%), we recorded low borderline handling method (BSMOTE and TL) occurrences across all data preparation pipelines (4% - 14%). This may have two reasons: first, some ML methods may be able to handle borderline cases more effectively compared to others, especially if they rely on kernel methods (e.g., SVM) or if they are ensemble approaches (e.g., RF, XGBOOST). And, secondly, the Tomek Links approach may overestimate the percentage of borderline samples, as it does not consider individual ML strategies to handle such samples.

The application of data preparation principles has been recently increasing in machine learning, radiomics and imaging analysis studies (50) (13, 24, 25, 46–48). These studies rely on manually pre-selected singular data preparation steps or combining thereof. Cysouw et al. performed dimensionality reduction by applying the principal component analysis (PCA); in addition, they performed subclass imbalance correction using SMOTE to characterize prostate cancer in [18F]DCFPyL PET (13). Umutlu et al. utilized least absolute shrinkage and selection operator (LASSO) regression to perform feature preselection, and in addition the employed adaptive synthetic (ADASYN) approach for subclass imbalance correction in their [18F]-FDG PET/MRI study to predict hormone receptor status and proliferation rate (48). Chang et al. employed SMOTE as subclass imbalance correction technique in their PET/CT radiomics study to predict anaplastic lymphoma kinase (ALK) rearrangement status in lung adenocarcinoma. In addition, they utilized LASSO regression for feature selection (46). Sanduleanu et al. employed recursive feature elimination (RFE) for feature selection, combined with SMOTE for subclass imbalance correction in their [18F]FDG-PET/CT radiomics study to predict tumor hypoxia (47). Parmar et al. investigated the effects of various feature selection algorithms combined with different ML classifiers to establish the highest performing predictive model for lung cancer and head and neck cancer cohorts (51, 52). Authors reported highest performing models of 0.69 AUC and 0.68 AUC respectively over independent validation data. Xie et al. investigated class imbalance correction approaches in a cohort of head and neck cancer patients in their [18F]FDG-PET/CT-based radiomics study, by testing various resampling techniques for generating minority subclass samples and for cleaning noisy and redundant data (25). Authors evaluated their preprocessed data using various classifiers, with highest reported performance increase of +0.32 AUC (0.50 AUC vs 0.82 AUC) with applying data resampling techniques. Their study utilized individual pre-processing methods, without combining them prior to machine learning. Furthermore, only Xie et al. compared predictive performance with and without data preparation (25). Lv et al. employed LASSO logistic regression for feature selection combined with various oversampling techniques for imbalance correction to predict lymph node metastasis (LNM) in clinical stage T1 lung adenocarcinoma (LUAD). The authors reported highest performance increase of +0.05 AUC (0.70 AUC vs 0.75 AUC) utilizing the edited nearest neighbors (ENN) method (53). Du et al. utilized various feature selection techniques combined with different classification algorithms for recurrence vs inflammation differentiation model establishment. The authors reported highest predictive performance of 0.89 AUC (0.83 sensitivity vs 0.87 specificity) utilizing the cross-combination of fisher score (FSCR) and random forest classifier (54). None of the above studies performed hyperparameter optimization of the utilized data preparation methods or automatized the building of data preparation pipelines.

Compared to the above studies, our proposed data preparation approach differs in several aspects: First, prior studies focused only on subclass imbalance correction and feature preselection, without handling outliers in their training data. Second, prior studies performed data preparation manually. This allows retesting various data preparation steps while utilizing the whole dataset prior to executing and reporting the given study, thus, may expose the given study to data leakage. In contrast, our approach provides a data preparation pipeline for the given data automatically, eliminating the possibility of data leakage that may occur due to incremental manual reuse of the whole dataset. Third, our solution provides hyperparameter optimization of various data preparation approaches being present in each pipeline on subsets of the training set.

The above findings imply that data preparation is indeed a non-trivial approach, however, it is a pre-requisite for state-of-the-art machine learning. Given the high level of expertise and the amount of time required to build optimal data preparation pipelines (27, 28), we argue that such activities may likely result in suboptimal prediction models, when performed manually. Given the above characteristics of our MLDP, it is applicable to a wide range of machine learning scenarios even beyond the scope of medical imaging. In contrast to the above, we wish to emphasize that MLDP does not substitute high-quality input data or clinical domain knowledge, which is still a prerequisite – even with utilizing MLDP – to properly collect, interpret and annotate data as well as to identify clinically-relevant hypotheses to be tested by building prediction models.

We also wish to highlight the relationship of our MLDP approach to automated machine learning (AutoML) approaches (30, 55). AutoML optimizes data preparation and ML classifier hyperparameters together. Therefore, in case of AutoML, the contribution of DP steps is not possible to analyze in retrospect. In contrast, our MLDP approach intentionally wishes to handle DP independently from ML, as it can better support the identification of biomarkers and the interpretation of data characteristics for clinicians before ML takes place.

Nonetheless, our study has a few limitations: First, only a limited number of preparation methods was included in the MLDP. Therefore, extending it with additional data preparation approaches may increase the resulting ML model performances. Second, we used default parameters for the utilized ML algorithms to build prediction models, while hyperparameter optimization (56) may further increase predictive performance.

## Conclusions

Automated data preparation (MLDP) can help increase the predictive performance of machine learning models, while eliminating the need of manual interventions to preprocess the data. Therefore, we consider that future machine learning studies, particularly in the field of clinical research shall rely on MLDP as a standard data preparation approach instead of performing such steps manually.

## Data availability statement

The raw data supporting the conclusions of this article will be made available by the authors, without undue reservation.

## Ethics statement

The studies involving human participants were reviewed and approved by Local Ethics Committee, Medical University of Vienna, Vienna, Austria. The patients/participants provided their written informed consent to participate in this study.

## Author contributions

Concept and design: DK and LP; Data acquisition: SR, NP, TT-W, ZR, HA, and AH; Data analysis/interpretation: DK, LP, CS, MG, and BE; Drafting of the manuscript: DK and LP; Critical revision of the manuscript: All; Statistical analysis: DK and LP; Administration, financial, or material support: TB and MH; Supervision: LP, TB, and MH; All authors contributed to the article and approved the submitted version.

## Conflict of interest

MH, LP, and TB are co-founders of Dedicaid GmbH, Austria.

The remaining authors declare that the research was conducted in the absence of any commercial or financial relationships that could be construed as a potential conflict of interest.

## Publisher’s note

All claims expressed in this article are solely those of the authors and do not necessarily represent those of their affiliated organizations, or those of the publisher, the editors and the reviewers. Any product that may be evaluated in this article, or claim that may be made by its manufacturer, is not guaranteed or endorsed by the publisher.
