# Supplementary material for: Automated data preparation for in vivo tumor characterization with machine learning
Source: Front Oncol. 2022 Oct 11;12:1017911. doi: 10.3389/fonc.2022.1017911 (PMC9595446; doi:10.3389/fonc.2022.1017911)
Supplement: Supplementary file 1 [file DataSheet_1.docx]

**Automated data preparation for in vivo tumor characterization with machine learning**

**SUPPLEMENTAL**

D. Krajnc^1^, C. P. Spielvogel^2,3^, M. Grahovac^2^, B. Ecsedi^1^, S. Rasul^2^, N. Poetsch^2^, T. Traub-Weidinger^2^, A. R. Haug^2,3^, Zs. Ritter^4^, H. Alizadeh^5^, M. Hacker^2^, T. Beyer^1^, L. Papp^1^

^1^QIMP Team, Center for Medical Physics and Biomedical Engineering, Medical University of Vienna, Vienna, Austria

^2^Medical University of Vienna, Department of Biomedical Imaging and Image-guided Therapy, Division of Nuclear Medicine, Vienna, Austria

^3^Medical University of Vienna, Christian Doppler Laboratory for Applied Metabolomics, Vienna, Austria

^4^University of Pécs, Medical School, Department of Medical Imaging, Pécs, Hungary

^5^University of Pécs, Medical School, 1st Department of Internal Medicine, Pécs, Hungary

Correspondence:

Thomas Beyer, PhD, MBA

QIMP Team, Center for Medical Physics and Biomedical Engineering, Medical University of Vienna, Vienna, Austria

Medical University Vienna

Währinger Gürtel 18-20

1090 Vienna, Austria

[thomas.beyer@meduniwien.ac.at](mailto:thomas.beyer@meduniwien.ac.at)

**Supplemental S1: Hyperparameters of utilized algorithms for outlier and borderline score calculation**

Table 1: List of hyperparameters for outlier and borderline score

| Outlier score | | Borderline score | |
| --- | --- | --- | --- |
| Algorithm | Isolation Forest | Algorithm | Tomek Links |
| Tree count | 1000 | Automatic | true |

**Supplemental S2: Pipeline tree generation restrictions and tree structure organization**

The restrictions in the pipeline establishbment were as follows:

1. If feature selection algorithm was already present in a pipeline, dimensionality reduction algorithm inclusion is disabled
2. If dimensionality reduction algorithm was already present in a pipeline, feature selection algorithm inclusion is disabled
3. Repeated inclusion of the algorithms from the same preparation group (e.g. oversampling) is disabled

Tree structure organization

In order to iteratively establsh a unique sequential lists of pre-processing methods for data preparation, all methods were organized in a tree structure, which is formed of tree root, tree nodes and tree leave. The tree represents all possible combinations of data pre-processing methods. (1-6) The path originating in the tree root and ending in the tree leaf describes an ordered list of data pre-processing algorithms to be performed sequentially over the training dataset. Each path in the tree is unique, therefore the creation of duplicates is exluded. See Figure 1 for the example of generated pipeline tree, where only one brach is visualized due to simplicity reasons.


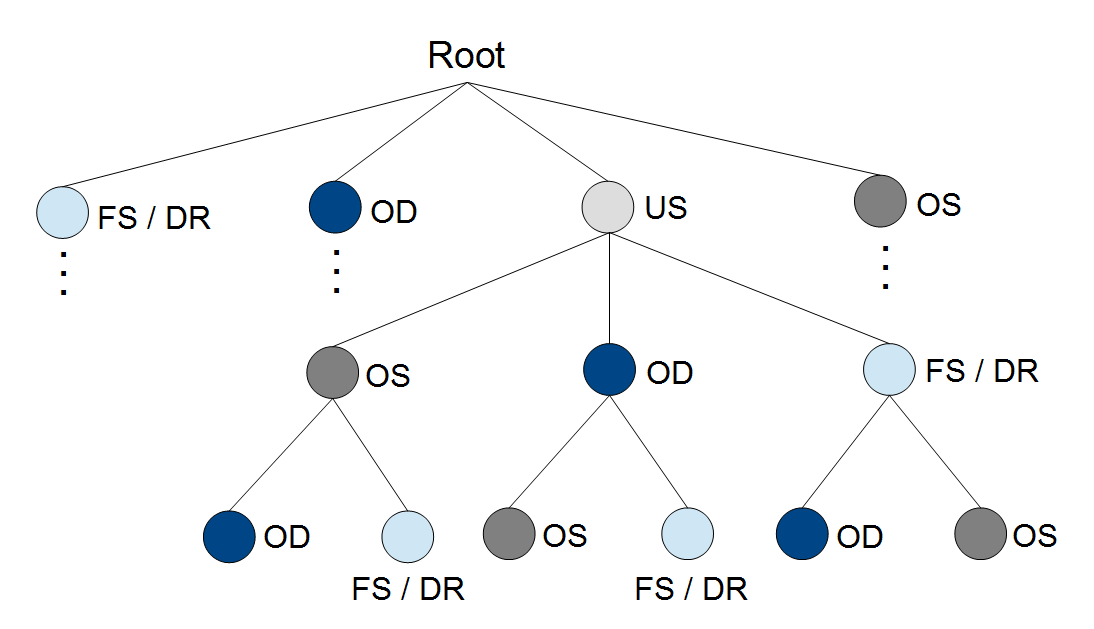


Figure 1: Pipeline tree example. FS – Feature selection; DR – Dimensionality reduction; OD – Outlier detection; US - Undersampling; OS - Oversampling;

**Supplemental S3: Evolutionary algorithm**

| Hyperparameter | Input value |
| --- | --- |
| Iteration count | 15 |
| Offspring count | 10 |
| Mutation rate | 0.6 |

The evolutionary algorithm (EA), which mimics the tournament selection, crossover and mutation (7) was employed to establish the highest performing set of data pre-processing methods (Figure 1). The EA is iteratively acquiring new sequential lists of preparation algorithms from the tree structure to increase the training fitness of the ML algorithm. The number of iterations is determined by the user-defined hyperparameter. See Figures 2a – 2c for the workflow of a single EA iteration for acquiring new data preparation pipelines. After all iterations are executed, the preparation pipeline with the best evaluated fitness is selected as optimal solution.


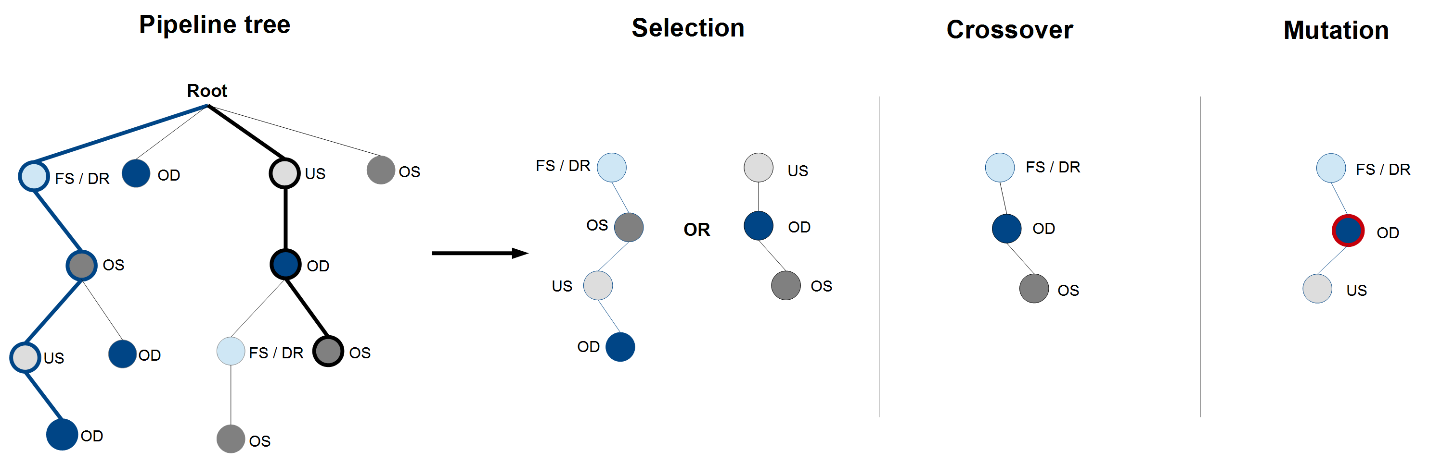


Figure 1: The example of data preparation pipeline (offspring) generation following the pre-established pipeline generation restrictions (Supplemental S2). Based on the pipeline tree, possible offspring types are generated by the action of tournament selection, crossover or mutation. FS – Feature selection; DR – Dimensionality reduction; OD – Outlier detection; US - Undersampling; OS - Oversampling;


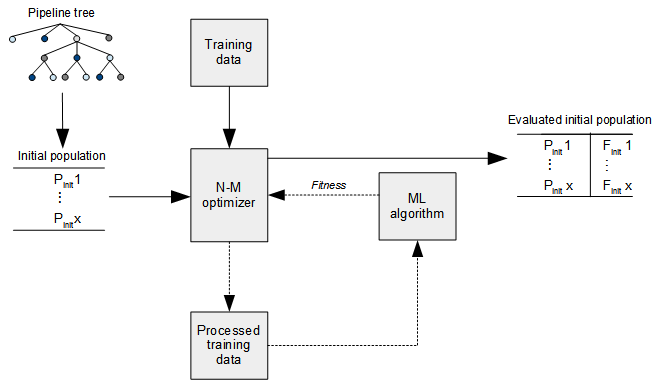


Figure 2.a: initial pipeline population establishment and fitness evaluation. The initial pipeline population is populated by importing the randomly selected pipelines from the pipeline tree. Each data prepration pipeline is provided as an input to nelder-mead algorithm for hyperparameter optimization, where each combination of hyper parameters is evaluated by internal machine learning algorithm over the training dataset. This process is engaged interatively until the optimal combination of hyperparameters is determined. Evaluated pipeline combined with calcualted fitness is stored in evaluated inital population.. P_init_ - initial pipeline; F_init_ – pipeline fitness; x – maximal number of pipelines (user-defined hyperparameter); N-M – Nelder-Mead; ML – Machine learning;


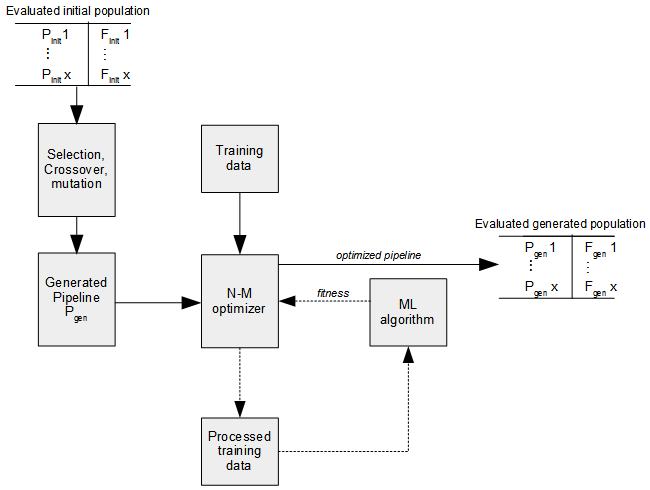


Figure 2.b: Generated pipeline population establishment and fitness evaluation. In each iteration, two pipelines from evaluated initial population are engaged with the evolutionary principles, where tournament selection, crossover or mutation may apply. The newly obtained pipeline is then evaluated by nelder-mead algorithm for hyperparameter optimization, as well as by internal ML algorithm for fitness calculation. Optimized pipeline combined with calcualted fitness is stored in the evaluated generated population. P_init_ - initial pipeline; F_init_ – pipeline fitness; P_gen_ - generated pipeline; F_init_ – pipeline fitness; x – maximal number of pipelines (user-defined hyperparameter); N-M – Nelder-Mead; ML – Machine learning;


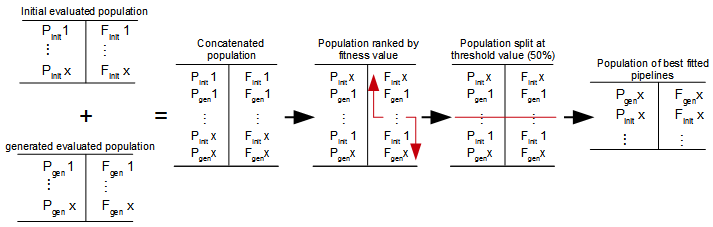


Figure 2.c: Concatenation, ranking and splitting of evaluated initial and generated pipeline population. After the fitness evaluation of both initial (n=x) and generated (n=x) population is performed (2a and 2b), evaluated populations are concatenated (n=2x). In the next step, all pipelines in the newly formed population are ranked by its respective fitness values. In the following step, ranked population is splitted by the treshold value (50%), keeping best fitted n=x pipelines. P_init_ - initial pipeline; F_init_ – pipeline fitness; P_gen_ - generated pipeline; F_init_ – pipeline fitness; x – maximal number of pipelines (user-defined hyperparameter);

Random forest (RF) (8) was employed as ML algorithm for fitness evaluation for all generated pipelines (Figure 2). The Nelder-Mead algorithm (NM) (9) was utilized for hyperparameter optimziation of each algorithm in the sequential list. Even though the MLDP utilized an internal random forest (RF) algorithm for the sole purpose of calculating an receiver operator characteristics (ROC) distance fitness value for each of its pipeline, the optimal pipeline can increase predictive performance in a wide-range of ML approaches as well. This is due to the fact that RF models are capable to model non-linear relationships in the data and they are inherently ensemble methods, that minimize the effect of individual model bias.

**Supplemental S4: Performance of all established ML prediction models**

Table 1: Performance evaluation based on confusion matrix analytics (CM) across all analyzed cohorts. ACC – Accuracy; SNS – Sensitivity; SPC – Specificity; MLDP – Machine learning data preparation; RF – Random Forest; MG – Multi gaussian; XGBoost – Extreme gradient boosting; NN – Neural networks; SVM – support vector machine;

|  |  | RF | | MG | | XGBoost | | NN | | SVM | |
| --- | --- | --- | --- | --- | --- | --- | --- | --- | --- | --- | --- |
| Cohort | Metric | *Without MLDP* | *With MLDP* | *Without MLDP* | *With MLDP* | *Without MLDP* | *With MLDP* | *Without MLDP* | *With MLDP* | *Without MLDP* | *With MLDP* |
| Prostate cancer | ACC | 0.78 | 0.78 | 0.74 | 0.75 | 0.79 | 0.79 | 0.77 | 0.78 | 0.77 | 0.77 |
|  | SNS | 0.73 | 0.76 | 0.72 | 0.72 | 0.8 | 0.79 | 0.83 | 0.77 | 0.8 | 0.76 |
|  | SPC | 0.81 | 0.8 | 0.75 | 0.77 | 0.79 | 0.78 | 0.71 | 0.78 | 0.74 | 0.78 |
|  | PPV | 0.78 | 0.78 | 0.72 | 0.73 | 0.77 | 0.76 | 0.72 | 0.75 | 0.73 | 0.75 |
|  | NPV | 0.77 | 0.80 | 0.76 | 0.76 | 0.82 | 0.81 | 0.83 | 0.79 | 0.81 | 0.79 |
| Glioma | ACC | 0.71 | 0.87 | 0.73 | 0.83 | 0.88 | 0.92 | 0.7 | 0.8 | 0.73 | 0.86 |
|  | SNS | 0.76 | 0.87 | 0.79 | 0.82 | 0.92 | 0.93 | 0.85 | 0.84 | 0.83 | 0.88 |
|  | SPC | 0.66 | 0.86 | 0.67 | 0.85 | 0.84 | 0.90 | 0.55 | 0.76 | 0.63 | 0.83 |
|  | PPV | 0.69 | 0.86 | 0.71 | 0.84 | 0.85 | 0.90 | 0.66 | 0.78 | 0.69 | 0.84 |
|  | NPV | 0.73 | 0.87 | 0.76 | 0.82 | 0.91 | 0.92 | 0.81 | 0.82 | 0.79 | 0.87 |
| DLBCL | ACC | 0.78 | 0.8 | 0.7 | 0.76 | 0.68 | 0.68 | 0.59 | 0.66 | 0.7 | 0.7 |
|  | SNS | 0.69 | 0.69 | 0.56 | 0.69 | 0.63 | 0.63 | 0.44 | 0.56 | 0.25 | 0.62 |
|  | SPC | 0.84 | 0.88 | 0.8 | 0.8 | 0.72 | 0.72 | 0.68 | 0.72 | 1.00 | 0.76 |
|  | PPV | 0.73 | 0.78 | 0.64 | 0.68 | 0.59 | 0.59 | 0.46 | 0.57 | 1.00 | 0.62 |
|  | NPV | 0.81 | 0.81 | 0.74 | 0.80 | 0.75 | 0.75 | 0.64 | 0.70 | 0.67 | 0.76 |

**Supplemental S5: Statistical analysis of ML predictive models with and without data preparation**

Table 1: Conventional statistical analysis of established ML predictive models with and without Machine learning data preparation (MLDP); RF – Random Forest; MG – Multi gaussian; XGBoost – Extreme gradient boosting; NN – Neural networks; SVM – support vector machine; σ – Standard deviation; CI – Confidence interval;

|  |  | Glioma | | Prostate | | DLBCL | |
| --- | --- | --- | --- | --- | --- | --- | --- |
| Model | Statistical method | Without MLDP | With MLDP | Without MLDP | With MLDP | Without MLDP | With MLDP |
| RF | mean ± σ | 0.71±0.15 | 0.865±012 | 0.77±0.04 | 0.79±0.04 | 0.76375±0 | 0.78375±0 |
|  | CI (95%) | 0.681 - 0.739 | 0.841 - 0.889 | 0.762 - 0.778 | 0.782 - 0.798 | 0.764 - 0.764 | 0.768 - 0.768 |
| MG | mean ± σ | 0.73±0.15 | 0.83±0.14 | 0.74±0.04 | 0.75±0.04 | 0.68125±0 | 0.74375±0 |
|  | CI (95%) | 0.701 - 0.759 | 0.803 - 0.857 | 0.732 - 0.748 | 0.742 - 0.758 | 0.682 - 0.682 | 0.744 - 0.744 |
| XGBoost | mean ± σ | 0.71±0.12 | 0.80±0.15 | 0.78±0.05 | 0.77±0.07 | 0.5475±0 | 0.63±0 |
|  | CI (95%) | 0.686 - 0.734 | 0.771 - 0.829 | 0.770 - 0.790 | 0.756 - 0.784 | 0.548 - 0.548 | 0.63 - 0.63 |
| NN | mean ± σ | 0.88±0.11 | 0.915±0.1 | 0.795±0.05 | 0.79±0.05 | 0.6725±0 | 0.6725±0 |
|  | CI (95%) | 0.858 - 0.902 | 0.895 - 0.935 | 0785 - 0.805 | 0.78 - 0.80 | 0.673 - 0.673 | 0.673 - 0.673 |
| SVM | mean ± σ | 0.73±0.15 | 0.86±0.12 | 0.78±0.04 | 0.77±0.07 | 0.625±0 | 0.6925±0 |
|  | CI (95%) | 0.701 - 0.759 | 0.836 - 0.884 | 0.772 - 0.788 | 0.756 - 0.784 | 0.63 - 0.63 | 0.693 - 0.693 |

**Supplemental S6: Hyperparameters of utilized data preparation algorithms**

Table 1: List of hyperparameters for data preparation algorithms. SMOTE – Synthetic minority oversampling technique; BSMOTE – Borderline synthetic minority oversampling technique; PCA – Principal component analysis; Auto parameter with value set to true allows full scale oversampling/undersampling utilization, achieving the 0% imbalance ratio. Only when set to false, oversampling technique may be instructed to up-sample the dataset by a certain percentage through the “oversampling percentage” parameter, or undersampling technique may be instructed to down-sample the dataset by a certain amount of samples through the “undersampling amount” parameter.

| Algorithm | Hyperparameters |
| --- | --- |
| Outlier detection | Tree count *(input number)* |
| Feature selection | Feature count *(input number)*  Rank method *(R-squared)* |
| Oversampling | Neighbors count *(input number)*  m_neighbors count *(input number)*  n_neighbors count *(input number)*  Auto *(true/false)*  Oversampling percentage *(if auto = false then input number)*  Type *(Random Oversampling, SMOTE, BSMOTE)* |
| Undersampling | Type *(Random Undersampling, Tomek Links)*  Auto *(true/false)*  Undersampling amount *(if auto = false then input number)* |
| PCA | Preservation percentage *(input number)* |

Note, that in this study undersampling and oversampling parameter auto=true was utilized which did not allow the mutual existence of Random Undersampling and any oversampling techniques in particular pipelines. The same was not necessarily true for e.g., Tomek Links and oversampling, as utilizing Tomek Links alone does not result in balanced datasets.

**References**

1. Marcano-Cedeno A, Quintanilla-Dominguez J, Cortina-Januchs MG, Andina D. Feature selection using Sequential Forward Selection and classification applying Artificial Metaplasticity Neural Network. IECON 2010 - 36th Annual Conference on IEEE Industrial Electronics Society2010. p. 2845-50.

2. Jolliffe IT, Cadima J. Principal component analysis: a review and recent developments. Philosophical Transactions of the Royal Society A: Mathematical, Physical and Engineering Sciences. 2016;374(2065).

3. Chawla NV, Bowyer KW, Hall LO, Kegelmeyer WP. SMOTE: Synthetic Minority Over-sampling Technique. Journal of Artificial Intelligence Research. 2002;16:321-57.

4. Nguyen HM, Cooper EW, Kamei K. Borderline over-sampling for imbalanced data classification. International Journal of Knowledge Engineering and Soft Data Paradigms. 2011;3(1).

5. Liu FT, Ting KM, Zhou Z-H. Isolation Forest. 2008 Eighth IEEE International Conference on Data Mining2008. p. 413-22.

6. T E, M A. Classification of Imbalance Data using Tomek Link(T-Link) Combined with Random Under-sampling (RUS) as a Data Reduction Method. Journal of Informatics and Data Mining. 2016;1(2).

7. Bartz-Beielstein T, Branke J, Mehnen J, Mersmann O. Evolutionary Algorithms. Wiley Interdisciplinary Reviews: Data Mining and Knowledge Discovery. 2014;4(3):178-95.

8. Sarica A, Cerasa A, Quattrone A. Random Forest Algorithm for the Classification of Neuroimaging Data in Alzheimer's Disease: A Systematic Review. Frontiers in Aging Neuroscience. 2017;9.

9. Lagarias JC, Poonen B, Wright MH. Convergence of the Restricted Nelder--Mead Algorithm in Two Dimensions. SIAM Journal on Optimization. 2012;22(2):501-32.
